# Mr. Stott, on Preparing Tartrit of Antimony

**Published:** 1804-06-01

**Authors:** Robert Stott

**Affiliations:** Surgeon. Dumfries


### *To the Editors of the Medical and Physical Journal.*

Gentlemen,

A Method of combining antimony and muriatic acid, by a double exchange in the dry way, by distilling a mixture of sulphurated antimony and corrosive sublimate, has been long known in chemistry, and the salt, resulting from this combination, has been named butter of antimony. Who was the author of this capital discovery, it is unknown to me, or, otherwise, I would do him the honour to acknowledge it; as it would shew him, whoever he was, to have been a chemist, at least, not inferior to some of those of modern times. There are, however, slight grounds to believe that it was known before the age in which Van Helmont lived. This author affirms that the alcahest of Paracelsus rendered metals distillable by the retort, and restored them again without alteration of their specific gravities; and, whether this may have any allusion to the distillation of butter of antimony, I leave it to the adepts to determine. The distillation of this butter of antimony is not attended with any visible discharge of noxious fumes, and may be conducted in the middle of a laboratory, in unluted vessels, without being offensive to the spectators, and with safety to the operator, if he only take care to tie a piece of cloth, or paper, round the upper part and neck of the retort, to retain the heat, and prevent the butter from congealing, and blocking up the nose of the retort, and exploding the vessels, till it drop down into the receiver. The butter, as it is called, when allowed to remain in an open vessel, exposed to a moist air, between three and five months, deliquesces into a clear liquid like water. In like manner, if the icy butter of antimony be distilled from a clean glass retort into a dry receiver, three times over, it becomes the liquid oil of antimony of the chemists. If the liquid oil, prepared in either of these ways, be dropt into water, a milkiness is immediately produced, and a white powder precipitates to the bottom of the vessel, which was called by the chemists, the angelic powder, the powder of algeroth, the mercury of life, &c. Within the last thirty years, Mr. Scheele, a German chemist, residing in Sweden, found that this powder dissolved readily in a solution of cream of tartar, at a boiling heat, and on evaporation furnished an excellent salt of antimony, greatly superior to the common tartar emetic which had had heretofore been in use among the chemists, and was always adulterated with heterogeneous salts. In order to save the great quantity of corrosive sublimate employed in preparing this powder, Mr. Scheele was induced to make trials, and actually did discover a method of preparing butter of antimony, viâ humidâ, without assistance of corrosive sublimate. As his process is conducted in an open digesting vessel, and gives out little fumes, it is perfectly safe to the operator, and as I have frequently tried it, and have always obtained more pulvis algerothi than half the weight of the antimony employed, I subscribe to the truth of it, always willing to give praise to whom praise is due.

Of late years, however, the Colleges of Physicians of Edinburgh, London, and Dublin, pretending, I suppose, to improve on Mr. Scheele's process, have published, in their Pharmacopœias, a process for muriat of antimony, which did not succeed with me; nor do I believe that ever it has succeeded, or ever will succeed, with any man, in its present form, for reasons to be presently mentioned. I have not learnt with certainty, whence this process originated. Certain, however, it is, that it has had a place for ten years in the Pharmacopœia of the Edinburgh College; and has, last year, been copied into the last edition of their Pharmacopœia; which circumstance renders it probable, that they have not seen their error during that time, and also that they have had no communication with the practical chemist; for, I doubt not, that many chemists, in this island, could have told them of their error, if they had been willing to hear it. From a circumstance which fell under my observation, I question whether they would listen to any representation on the subject, much less thank any one for his communication. Till I saw it again honoured with a place in the Edinburgh Pharmacopœia, and Dispensatory, I surely thought their process was long ago exploded; and I think I may safely advance, that if the Colleges had been under necessity of preparing the powder of algeroth for the use of their patients, or in the way of the practical chemist, it never could have had a place in any Pharmacopœia. The Editor of the Edinburgh Dispensatory, in 1794, has given this mode of preparing muriat of antimony a preference to every other mode of preparing it, although he has been guarded enough not to point out wherein its superior excellency consists; but the Editor of the same work in 1803, has written of it with more reserve,

Having

Having thus observed, that the error was become general, and might be a means of misleading the young chemist, and of damping his juvenile ardour, at the loss of his vessels and materials, and might in this way tend to retard the progress of chemistry, I thought it proper to communicate the result of my experiments to the public, though perhaps, I may be little attended to in a question between me and three Collegiate bodies of Philosophers.

The oxyd of antimony with sulphur, by nitrat of potash, as the Edinburgh College is pleased to call it, which is a preliminary step to the distillation of muriat of antimony, is prepared by deflagrating in a red hot crucible, equal weights of sulphurated antimony and of nitrat of potash, thoroughly mixed and dried. The oxyd thus obtained is to be pounded and washed repeatedly with boiling water, and dried. A pound of sulphuric acid, which is not seven ounce measures, is next to be put into a retort, and afterwards there is to be added, by degrees, one pound of the above oxyd of antimony, previously mixed with two pounds of the dried muriat of soda. This mixture is to be distilled in a sand bath, the distilled matter is to be exposed to the air for several days to relent or deliquesce into a liquid, and the fluid part poured off from the dregs. Now, let facts speak for themselves. I proceed to the experiments.

Feb. 20, 1797, Having prepared oxyd of antimony, with equal weights of nitrat of potash and of antimony, I washed it with boiling water repeatedly, then dried it, and found that two pounds and a half, from the crucible, lost one pound by this treatment. The oxyd was now of a dark red or brown liver colour. Twenty-four ounces of it, mixed with an equal weight of dried muriat of soda, were put into a large retort. Nearly sixteen ounces of sulphuric acid were next poured upon this mixture, and the retort set into a sand-bath. No sooner did the sulphuric acid touch this mixture, than the muriatic acid began to pass away copiously, in white suffocating incondensible vapours, and with a smell of sulphurated hydrogen gas (a liver of sulphur smell.) A capacious receiver was applied, with a hole in the side for applying a syphon, at which, during the distillation, the muriatic acid carrying some little oxyd of antimony along with it, passed away in a continued stream, and contaminated the laboratory so much, that no person could stay in it, notwithstanding a draught of air through it. The phenomena were such that I durst not lute the vessels completely for fear of explo-

 sion; sion; and indeed this seemed the less necessary, since in the old process for muriat of antimony by muriat of quicksilver, no luting is required. This was one of the most intolerably suffocating operations I ever performed. After the mixture distilled to dryness, on lifting out the retort, I observed that the sulphuric acid, for want of fluidity, as we may say, had not penetrated the half of the mass, and much of the oxyd lay unchanged, but compacted into a firm lump which could not be dissolved, or be got out without breaking the retort. A thin crust was collected about the neck of the retort, of a deep yellow colour, which did not liquefy by heat, like muriat of antimony, nor could it be got off, in any other way, to be of use. It extended downwards to nearly the surface of the residue. After all, the quantity of muriat of antimony, if such it might be called, obtained in the receiver, both in a fluid form, and sticking to the sides of it, in form of a yellow crust, not liquefiable by heat, was a mere trifle. After precipitating it with water and washing it thoroughly, I obtained twelve drachms and thirty-six grains of powder of algeroth, out of twenty-four ounces of red oxyd of antimony, which I began with; which is little more than one ounce out of sixteen ounces, and not an eighth part of what could have been got by Mr. Scheele's process. But it is to be observed, that the yellowish crust on the receiver, could not be completely washed off, but still left a yellow stain. The mass was afterwards pounded and treated with muriat of soda and sulphuric acid, via humida, and this repeated, by which I obtained more muriat of antimony; but still a great quantity of black matter remained, which was sulphurated antimony unchanged. It seemed to be about a pound, but I did not weigh it, and was further insoluble in muriatic acid. The first observation that naturally occurs to one on perusing the account of my experiment, is, that I have inverted the order of proceeding, recommended by the Colleges, in adding the sulphuric acid to the dry materials; whereas, they order the dry materials to be added, by degrees, to the sulphuric acid. To this I answer, the method of proceeding, ordered by the Colleges, is evidently copied from Mr. Scheele's process, where the operation is conducted in an open digesting vessel; the diluted sulphuric acid is first allowed to dissolve the sulphat of potash, and combine with the oxyd, and afterwards quits it, when the muriat of soda is presented, in consequence of a double exchange; the sulphuric acid combining with the soda, and the the muriatic acid with the oxyd of antimony. But here, as the process is done in a retort, the dry materials cannot be added, by degrees, to the sulphuric acid, without some of them sticking to the neck of the retort, (unless done in a tubulary one) and besides, after the addition, the fumes issue out so copiously that the operator is obliged to look to his own personal safety; to add the whole as quick as possible, and run. In the second place, I used only an equal quantity of muriat of soda and of oxyd of antimony, although the Colleges order a double quantity of the former; but this I had previously found, from trials in Mr. Scheele's way, to be sufficient to dissolve the whole of the oxyd in an equal weight of crocus of antimony. Thirdly, I used rather a larger proportion of sulphuric acid, in order if possible to save my retort, but in vain. And here I think it worthy remark, that the Colleges certainly had not foreseen that twelve ounces of sulphuric acid, which are not equal to seven ounce measures, were not sufficient to wet even the half of one pound of oxyd of antimony and two pounds of dried muriat of soda, and, therefore, that it was impossible that the necessary combinations could take place, even if the materials had been in a proper state for it. The fourth observation I shall make on this process, is, that the muriat of antimony is supposed to be in a solid form, like butter of antimony of the chemists, since it is to be exposed to the air for several days to attract humidity, and dissolve into a liquid; and, even this circumstance shews that the Colleges have never tried their process, for in my experiment the small quantity of muriat of antimony obtained, was in a fluid form, and that only in consequence of the small quantity of water in the sulphuric acid. Finally, the Colleges have not made any provision for the condensation of the loathsome suffocating vapours, nor for saving the retort; and this last consideration, even if muriat of antimony could be obtained by it, would give Mr. Scheele's process a decided preference with the practical chemist.

As a mistaken theory has led to this process, I shall point out, as nearly as I can, to what causes the want of success is to be attributed. It seems to me to depend chiefly on two circumstances. First, the antimony is not oxydised sufficiently, as clearly appears from the experiments above, where, after all that muriatic acid could dissolve, a great quantity of antimony was brought into view, seemingly unchanged; but this was pointedly enough taken notice of by that excellent chemist, Mr. Scheele.—

The oxydation of antimony, too, has its bounds; for, if it be oxydised white, not a particle of it will dissolve in muriatic acid, as I found at the expence of a considerable quantity of materials.

The second circumstance, to which the want of success is to be attributed, is the form of applying the muriatic acid; a proper quantity of fluid not being present as a medium through which the ingredients may act on one another. An opinion has been entertained by many chemists, since the time of Boerhaave, (see Boerhaave's Elements of Chemistry, sect. ix. on neutral salts considered as menstruums) that muriatic acid, in a concentrated form, was capable of dissolving metallic bodies, on which it could produce no effect in a watery state. This inference they have drawn from a few experiments, in the way of cementation and sublimation, conducted with sulphat of iron and muriat of soda. All my experiments, however, concur to shew, that muriatic acid, whether concentrated or diluted, acts but weakly upon metallic bodies, in general, so long as it remains common muriatic acid, and produces no sensible effect whatever either upon antimony or quicksilver; but no sooner does it meet with manganese, or red oxyd of iron, (vulgo colcothar) than it becomes oxygenated, and presently oxydizes and dissolves metals, on which it exerted no power before; and, it appears to me, that its action, under this modification, can be extended much farther in a watery than in a dry concentrated form, merely by protracting the digestion; whereas, in the dry way, it has not time to act to the same advantage, being forced off with strong heat. And oxyd of iron being present in all the operations done with sulphat of iron, explains the effects, falsely attributed to the concentrated muriatic acid. But, even if concentrated muriatic acid were required in this process, it could not be applied to advantage to any metal, by pouring sulphuric acid upon dry muriat of soda.

In the experiments alluded to above, with the sulphatic salts, the muriatic acid is disengaged in a slow progressive manner, in the way of exchanges; but no sooner is sulphuric acid poured upon dry muriat of soda, than the muriatic acid is forced off instantly, in white vapours, and passes away without shewing the least disposition to condense. Even, if a quantity of dry muriat of soda be put into the bottom of a tall vessel, a high column of water poured gently over it, and the sulphuric acid poured thro' the water upon the salt, a considerable portion of the mu-

2 riatic riatic acid, instantly expelled with noise, will rise up thro' the water, and fly off, before the operator can agitate the mixture. Accordingly, all expert chemists have found it expedient to dilute the sulphuric acid, in distilling muriatic acid from muriat of soda; and when matters are thus managed, the work goes on quietly, and the vessels may be luted from the beginning. Indeed, Glauber, to whose industry we are indebted for this method of distilling muriatic acid, and, after him, Boerhaave, sometimes used sulphuric acid without dilution; but, in this case, they kept their vessels open till the volatile acid made its escape, and, when they used bole or brick-dust, in place of sulphuric acid, they previously expelled the wild crackling spirit, as they called it, by decrepitation; by which uneconomical mode of proceeding they always lost a great deal of the muriatic acid.

I have thus, I trust impartially, delivered an opinion, grounded on experiments, concerning the process of three Colleges of Physicians for preparing muriat of antimony, and I wish it to be understood, that in what I have advanced, I may be considered as having addressed myself chiefly to the practical chemist. To those who form their opinions, in these matters, according to the degree of credit they place in this or the other writer, or body of men, I would say little, as it would avail little, till they have learnt something of the practice of chemistry. With the former, my arguments will have due weight, as in every trial of this kind his vessels and materials are at stake; and, if he entertains any doubts of my veracity, he has only to light up his furnace, and repeat the experiment; a single instance will convince him of what I have advanced.

I shall now subjoin an account of Mr. Scheele's process, and of the method of properly preparing tartrit of antimony by it.

Take of antimony one pound, of nitrat of potash one pound and a half; mix and dry them, and deflagrate in a crucible or iron mortar. Pound the oxyd thus obtained, and put one pound of it into a glass matrass or digester, and pour upon it fifteen ounces of sulphuric acid, diluted in three pounds of water, and afterwards add fifteen oz. of muriat of soda. Digest, Mr. Scheele says twelve hours, but I have found two or three hours, in general, sufficient, and during that time the mixture must be frequently stirred, and the heat kept moderate, for the muriat of antimony will begin to evaporate before it boils. The muriat of antimony, when completely formed, which is known by the milkiness of the solution, is to be strained through linen, and diluted with boiling water, that the pulvis algerothi may precipitate, which is to be repeatedly washed and dried. The pulvis algerothi, thus prepared, still retains a portion of muriatic acid, which cannot be separated from it by ablutions; for, after having washed it upwards of twenty times, in fresh portions of distilled water, I observed that the last had as much of the rough, harsh taste of muriatic acid as the first. But I have never been anxious about separating the muriatic acid entirely, in the suspicion that I might thus alter the state of the oxyd; a circumstance that ought to be carefully guarded against, a slight degree on either side rendering it insoluble in muriatic acid or in acid of tartar. Besides, since I followed Mr. Scheele's method, I have been of opinion that an under proportion of muriatic acid was essential to the constitution of good tartrit of antimony.

With oxyd of antimony, prepared by heat, or by nitrat of potash, I never could obtain tartrit of antimony in transparent crystals; they were always opaque, and stained orange, yellow, or green; a sure mark of an incomplete combination; whereas tartrit of antimony, from pulvis algerothi, precipitated by water, concretes into transparent crystals, which become rather white, or effloresce a little in the open air. In preparing tartrit of antimony, I put nine ounces of acidulous tartrit of potash into a matrass with twelve pounds of water, and when the solution boils, add, by degrees, about six ounces, or six ounces and a half, of pulvis algerothi. During each addition, I have observed a kind of effervescence excited. The quantity of water here ordered, is not sufficient to dissolve, with boiling heat, the acidulous tartrit of potash; but as the tartrit of antimony forms, which is a very soluble salt, the whole comes to dissolve gradually, and thus a considerable time is saved in the evaporation. I am convinced that even less water would suffice, for the reason now mentioned. When the whole is added, the solution is allowed to boil about two hours, perhaps less time would suffice, but as the pulvis algerothi is generally mixed with a grain or two of sulphur of antimony, which passes the filtre, one cannot see when the solution is complete. On this account, the vessel should be allowed to stand in the surface of the sand till the powder subsides, when the acid of tartar will be found to be completely saturated, and a little pulvis algerothi at bottom, mixed with the sulphur of of antimony now mentioned. It is this dark-coloured powder which yields a smell of sulphurated hydrogen gas (liver of sulphur smell) on boiling, for after the solution is passed through a filtre of paper, and again evaporated, the smell is no more observable.

After the solution is boiled down considerably, I pour it into a bason, and again evaporate with a heat gradually decreasing through the night, and next morning I have generally the satisfaction to observe, after breaking the pellicle, fine crystals of tartrit of antimony on the bottom of the bason. The solution must be decanted off into another bason, for further evaporation. The crystals, when allowed to dry in a gentle heat, and examined, I have found to be pyramids, with four flat sides and four right angles, joined together by their bases, and thus the pyramid of one end forming a parallelopipedon with the pyramid on the other end. A very thin square table or plate intervenes between the bases of the two pyramids. Some of the pyramids I have observed to have two of their opposite sides broader than the other two opposite sides, thus exhibiting the appearance of a wedge. The tartrit of antimony thus prepared, though tasteless, still shews signs of muriatic acid, joined to oxyd of antimony, for on scraping the crystals from the bason with a clean polished knife, I found it affected in the same way as from calomel; the muriatic acid readily preferring iron to antimony or quicksilver. Hence, it is a kind of quadruple salt, consisting of oxyd of antimony with muriatic acid, rendered soluble by acid of tartar, combined with an under proportion of potash. And here I think it worthy remark, that I have not found a name, in the new Nomenclature, expressive of its constituent parts.

[To be continued.]

ROBERT STOTT,
Surgeon.

*Dumfries, April* 3, 1804.

